# Sleep fluctuations precede self-reported mood changes in bipolar disorder: results from the BipoSense study

**DOI:** 10.1186/s40345-026-00416-y

**Published:** 2026-03-27

**Authors:** Andrea Ulrichsen, Esther Mühlbauer, Vera Miriam Ludwig, Emanuel Severus, Anthony Cleare, Sameer Jauhar, Michael Bauer, Ulrich W. Ebner-Priemer

**Affiliations:** 1 Department of Psychiatry and Psychotherapy, Dresden University of Technology, University Hospital, Dresden, Germany; 2 Institute of Psychiatry, Psychology and Neuroscience, Department of Psychological Medicine, King’s College London, London, UK; 3 Mental mHealth Lab, Institute of Sports and Sport Sciences, Karlsruhe Institute of Technology, Karlsruhe, Germany; 4 Department of Psychiatry and Psychotherapy, Central Institute of Mental Health, Medical Faculty Mannheim, University of Heidelberg, Mannheim, Germany; 5 Department of Brain Sciences, Faculty of Medicine, Imperial College London, London, UK

**Keywords:** Bipolar disorder, Sleep and mood change, Symptom prodromes, Early warning signs, Sleep and mood diary, Longitudinal study design

## Abstract

**Background:**

The temporal relationship between sleep and mood changes in bipolar disorder (BD) has been investigated before, and this paper aims to replicate results from previous analyses while adding new details to the understanding of the relationship between fluctuations of sleep and mood. Furthermore, we comment on the use of sleep changes as a prodrome to mood changes in BD, which could improve clinical outcomes.

**Methods:**

BD outpatients in remission (*N* = 29) recorded daily their sleep of the past 24 h and rated their mood on a visual analogue scale for 1 year (total of 9,433 days). Cross-correlation functioning was employed to identify potential relationships between self-reported sleep values and mood scores, for both the days before and after a change in mood.

**Results:**

41% of participants reported a negative relationship between changes in total time spent in bed and mood the following day, e.g. spending more time in bed before a shift towards depressive symptoms. Additionally, 21%-28% of all participants experienced an increase (or decrease) in their 7-day sleep average (sleep duration and awake in bed duration) in the week before a change in mood towards a lower (or higher) score. Only a few participants showed any relationship between changes in the 7-day variability of sleep and mood change.

**Conclusion:**

Our findings align with and support those of earlier studies with similar designs. The duration of sleep and time in bed may serve as early indicators of mood changes in BD for about two-fifths of patients, and integrating these symptoms into clinical practice may help anticipate critical clinical shifts.

## Background

Bipolar Disorder (BD) is a chronic mental illness characterised by fluctuations between depressive, hypomanic/manic and euthymic mood episodes (McIntyre et al. 2020). Symptoms of BD not only include low, irritable, or elated moods, but also an increased need for sleep, insomnia and sleepiness when depressed, as well as increased energy and lower sleep need when in hypomanic/manic episodes (American Psychiatric Association 2022). In addition to the symptom burden, patients experience reduced psychosocial functioning and up to 20 years shorter life expectancy, primarily due to cardiovascular diseases and suicide (McIntyre et al. 2020).

People with BD usually experience sleep issues throughout their lifetime, even in phases of euthymia, which affects their quality of life (Jermann et al. 2022). A study of 61 BD patients found that quality of life was correlated with insomnia complaints, sleepiness, and depressive symptoms, and suggested that focusing on improving sleep quality would help improve general quality of life (Jermann et al. 2022). Apart from its role in quality of life, sleep is also thought to have an essential role in emotional regulation and, therefore, the core symptomatology of BD (Morton and Murray 2020).

The recurrence of BD episodes is often unpredictable, and identifying prodromes, i.e. any signs or symptoms occurring before changes in mental health status (Andrade-González et al. 2020), could hold an important key in the management and treatment of BD. Known prodromes include increased energy and activity (Andrade-González et al. 2020), use of antidepressants (Rodrigues Cordeiro et al. 2023) and sub-clinical mood changes (Jackson et al. 2003) before manic episodes, and stressful life events (Rodrigues Cordeiro et al. 2023) and loss of interest (Andrade-González et al. 2020) before depressed episodes. Notably, a systematic review looking into people’s own identified changes before mood episodes found that up to 90% (median 70%) of people with BD experienced some form of sleep disturbance before a hypomanic/manic episode, and although fewer people identified it before a depressive episode (median 24% ) (Jackson et al. 2003), sleep changes before a depressed episode have also been found in other studies (Andrade-González et al. 2020, Rodrigues Cordeiro et al. 2023). The nature of the sleep changes, however, remains unclear.

Daily data collection is a valuable tool in identifying changes in symptomatology that might otherwise have been overlooked by either patient or clinician (Bauer et al. 2023). A recent systematic review (17 publications, 1322 BD patients) looked at the specific sleep disturbances identified before changes in mood in BD (Ulrichsen et al. 2024) using daily collected data for a minimum of 3 weeks. The BD patients experienced changes towards longer sleep duration, falling asleep earlier and waking up later before identifying increasing depressive symptoms. The opposite was found for mania with shorter sleep duration. These changes in sleep patterns appeared to be predictive of specific mood polarity and could potentially be used as prodromes for not only changes in symptomatology but also mood episodes (Ulrichsen et al. 2024). Some studies have found that, in particular, variability values over several days, not mean values, are predictive of episode onset (Ulrichsen et al. 2025) or symptom severity (Kaufmann et al. 2016). However, there still appears to be gaps in the literature on the correlation between sleep and mood. For example, although many of the studies included in the review looked at the direct correlation between daily ratings of sleep and mood (Ulrichsen et al. 2024), many lacked nuances in sleep data collected (e.g. only looking at sleep duration and time spent awake in bed) (Dominiak et al. 2022, Melbye et al. 2021, Leibenluft et al. 1996), or had a short study duration and therefore not allow for reliable long-term trends, which is important in a chronic illness (Bauer et al. 2023). Of those studies that did look long-term (> 3 months study duration), many had poor response rates and a median study duration for participants of < 3 months, due to patients not being followed for the full study length (Melbye et al. 2021, Tseng et al. 2022).

Given the potential clinical utility of using sleep changes in the prediction and early detection of mood episodes, with a continuing overall lack of clear evidence, we believe this work warrants further exploration. Prior research by Bauer et al. (2006), Bauer et al. 2008) has informed the present study’s methodological approach, examining daily sleep data and its correlation with mood changes. Their longitudinal design incorporated not only sleep duration but also time spent awake in bed, as well as the combination of the two. This combined value was proposed as a potentially more reliable data entry in a self-reported data-set, given it might be easier to recollect time going to bed and getting up, rather than time falling asleep and awaken (Bauer et al. 2006). Furthermore, the analysis used - individual time series analysis - is appealing because it revealed the number of participants showing significant relationships between sleep patterns and symptomatic shifts. Clinically, this provides more insight than a group effect, which does not clearly indicate how many patients actually exhibit the reported pattern.

This publication aimed to investigate the relationship between fluctuations of sleep and mood in BD in a longitudinal observational study over 1 year using data from the BipoSense study (Ebner-Priemer et al. 2020). More specifically, we investigated whether daily changes in sleep variables co-occur with or precede changes in self-reported mood over a period of up to 7 days. The primary objective of this paper was to replicate the data analyses presented by Bauer et al. (2006), using both sleep duration, awake in bed, and total time spent in bed as the sleep variables. The second objective was to expand on the analyses by Bauer et al. (2006) by exploring sleep averages over 7-day periods, as well as the role of sleep variability (SD) over 7 days, and the correlation with daily mood changes. Since previous analyses from the BipoSense study focused on shifts in clinical mood episodes (Ulrichsen et al. 2025), these additional analyses would enable us to assess changes in sub-clinical mood symptoms and comment on the differences in predicting mood episodes versus mood symptoms. All analyses were exploratory and not preregistered.

## Method

### Study protocol

The BipoSense study was a longitudinal study collecting daily sleep and mood data from BD participants over one year (Ebner-Priemer et al. 2020). It was approved by the local ethics committee at the Medical Faculty of the Technical University of Dresden (EK-Nr.: 26012014) and adhered to the Declaration of Helsinki. All participants included in the study provided written informed consent to participate. This is a secondary data analysis of the data collected from this study.

### Study design

The study design of BipoSense has previously been described (Ebner-Priemer et al. 2020). In short, BD patients were recruited through a specialised outpatient clinic from Dresden University Hospital, Germany. Criteria for inclusion were 1) BD I or II diagnosis (as assessed by SCID-I) in remission, which was defined as YMRS (Young Mania Rating Scale) (Young et al. 1978) score ≤ 12 and MADRS (Montgomery and Asberg Depression Rating Scale) (Montgomery and Asberg 1979) score ≤ 12; 2) ≥ 3 affective episodes within the past five years (≥ 1 being (hypo)manic); 3) ≥ 18 years old; and 4) willing and able to use a smartphone. Participants were excluded if they had substance abuse (not including caffeine or tobacco), comorbid personality disorder, neurological disorder, or other clinically relevant physical illnesses.

### Study procedures

After enrolment, participants underwent clinical interviews with trained psychologists every two weeks over the course of one year to assess changes in mood episodes. This data is not included in the analyses conducted for this manuscript. Additionally, participants were asked to use the movisensXS app (movisens GmbH, Karlsruhe, Germany) daily to report their sleep and mood. The items asked were adapted from ChronoRecord, a validated and previously used tool to collect daily mood scores and sleep data (Bauer et al. 2008, Bauer et al. 2004). By the end of every day, participants recorded their overall mood on a visual analogue scale ranging from 0 (depressed) to 100 (elevated), with 50 indicating “even-tempered” or neutral mood. Sleep patterns were recorded by selecting one of three icons (awake, asleep, or awake in bed) for each hour over the past 24 h. In addition, patients recorded any medication they had taken and mobile sensing-parameters were monitored (Ludwig et al. 2025), however not included in the presented analyses. Participants were prompted by the app multiple times each evening to complete their end-of-day e-diary. The day was recorded as missing if they did not respond by 23:59. All participants received 35 euros per month in compensation for their time, travel expenses, and mobile phone contracts. They used their own smartphones or got a study phone.

### Data preparation and analysis tools

Data preparation and analyses were conducted using IBM SPSS Statistics (version 29.0.1.0). The analysis techniques were used in the original publication (Bauer et al. 2006) and aimed to identify correlations between sleep variables and mood scores. SPSS code and strategy can be requested from the corresponding author (AU).

### Time series and cross-correlations

Time series were calculated for each participant across four variables: sleep duration, awake in bed duration, total time in bed, and mood. To model individual trends and filter out noise in the data, the Auto-Regressive Integrated Moving Average (ARIMA) methodology (Diggle 1996, Box et al. 2015) was applied. Specifically, an ARIMA (0,1,1) model was employed, incorporating a first-order moving average and first-order differencing. This approach eliminates linear trends and captures day-to-day shifts in the data, making it suitable for the analysis and results presented in this paper. Each ARIMA model generated both predicted values, representing the expected patterns, and residuals, defined as the difference between the predicted and actual values. Thus, the residuals captured the unanticipated or unexplained changes in the time series.

The Cross-Correlation Function (CCF) was applied to the residuals to identify linear relationships between self-reported sleep variables and mood scores, across time lags ranging from ± 7 days. By focusing on the residuals, the CCF analyses targeted sudden or unexpected changes in sleep and mood —that is, the changes not accounted for by trends identified in the ARIMA models. CCF was calculated separately for each participant to provide the Standard Error (SE) of the correlation coefficients for each defined lag comparison of sleep variables with mood assessment. Lag = 0 indicates the CCF between the unexpected change in sleep and mood recorded on the same day; however, as both were recorded by the end of the day, the values represented by lag = 0 are actually sleep values from the night before and mood on the current day. Significant results were indicated by correlations of two Standard Errors (SE) above or below zero (± 2*SE). Relationships can be positive (when sleep and mood change in the same direction) or negative (when sleep and mood change in opposite directions). The results are summarised by tallying the CCF results from all participants, showing how many participants had a significant CCF over specific days between fluctuations in sleep variables and mood change.

### Additional analyses

Moving Averages (*mA*) over the past 7 Days and Moving Standard Deviations (*mSD*) fitting to the *mA*s (of the past 7 days) were calculated for sleep variables over 7-day periods to explore their correlation with mood. These are not the same moving averages explained above in the ARIMA model, but instead new calculations for each day, based on the previous 7 days. These analyses provided insights into how average sleep patterns and their variability over a week correlated with daily mood changes, investigating whether more extended periods of sleep changes were perhaps a better predictor of mood change.

The CCF analyses were conducted according to the original analyses by Bauer et al. (2006) using the residuals from the time series of sleep duration (hours asleep), awake in bed duration (hours awake while in bed) and total time in bed (combined time spent in bed, awake or asleep). Additionally, we calculated the moving average (*mA*) and moving SD (*mSD*) values of these three sleep variables of the last 7 days, and used these time series for our new analyses: *mA* Sleep duration, *mA* awake in bed duration, and *mA* total time in bed, as well as *mSD* Sleep duration, *mSD* awake in bed duration, and *mSD* total time in bed.

## Results

### Participants

53 patients were screened for participation, and of those, 31 met the required inclusion criteria. 2 of those were subsequently excluded due to technical errors. A total of 29 participants were included in the study. The sample mean was 44 years old (SD = 11.9, range 25–70), with 55% of the participants being female (16 females and 13 males). Additionally, 59% were diagnosed with BD-I (17 BD-I/12 BD-II). On average in their lifetime, the participants had experienced depression 7.1 times (SD = 5.6), hypomania 3.0 times (SD = 3.8), and mania 2.8 times (SD = 3.5), and were hospitalised for BD 3.6 times (SD = 3.7, range 0–15). Participants completed 10,587 study days (mean per participant = 365, range 308–398 days) with excellent compliance, as evidenced by 97% (*N* = 726) of the planned clinical interviews being completed and 89% (*N* = 9433) of the daily e-diaries being filled out. See Table 1 for details.

**Table 1:** Demographic and clinical baseline data

-	-	*N* (%)	Mean	Minimum	Maximum	SD
Demographic	Age		44	25	70	11.9
	Gender	Female: 16 (55.2)				
	BD-I	17 (58.6)				
	BD-II	12 (41.4)				
	Lifetime depressive episode		7.1	2	30	5.6
	Lifetime hypomanic episode		3	0	15	3.8
	Lifetime manic episode		2.8	0	10	3.5
	Lifetime hospital admissions (BD)		3.6	0	15	3.7
	MADRS score		2.9	0	10	3.3
	YMRS score		1.2	0	8	2.1
	BRMRS score		0.7	0	6	1.4
Medications*	Lithium	15 (51.7)				
	Atypical antipsychotics	10 (34.5)				
	Anticonvulsants	11 (37.9)				
	Antidepressants	8 (27.6)				

Baseline characteristics of the 29 participants. SD = Standard deviation; BD = Bipolar Disorder; MADRS = Montgomery and Asberg Depression Rating Scale, YMRS = Young Mania Rating Scale; BRMRS = Bech Rafaelsen Mania Rating Scale. * Participants may be taking more than one type of medication

### Sleep and mood

During the study, participants slept on average 7.9 h per night (SD = 1.2) and were awake in bed 0.7 h per night (SD = 0.9). Participants reported an average mood of 48.7 (aggregated mean), spent 45.2% of their time with scores less than 50 and 50.8% of their time with scores more than 50. In total, there were 30 depressive episodes and 20 (hypo)manic episodes. See Table 2 for details.

**Table 2:** Sleep and mood data over the course of the study

-	-		*N*	Mean (aggregated)	Min	Max	SD
Sleep data	Hours awake			15.3	12.9	17.2	1.0
	Awake in bed (hours)			0.7	0.1	3.6	0.9
	Sleep duration (hours)			7.9	4.8	10.3	1.2
Mood data	Self-reported mood			48.7	24.0	58.8	6.5
	Time spent with self-reported mood less than 50 (%)			45.2%			
	Time spent with self-reported mood more than 50 (%)			50.8%			
	Number of depressive episodes		30				
	Number of (hypo) manic episodes		20				

Aggregated mean/min/max (of participant-means) of sleep and mood data collected during the study. The number of depressive and (hypo)manic episodes is based on clinical interviews. SD = Standard deviation; Min = Minimum participant mean; Max = Maximum participant mean

#### Daily fluctuations of sleep and mood changes

**Fig. 1:**
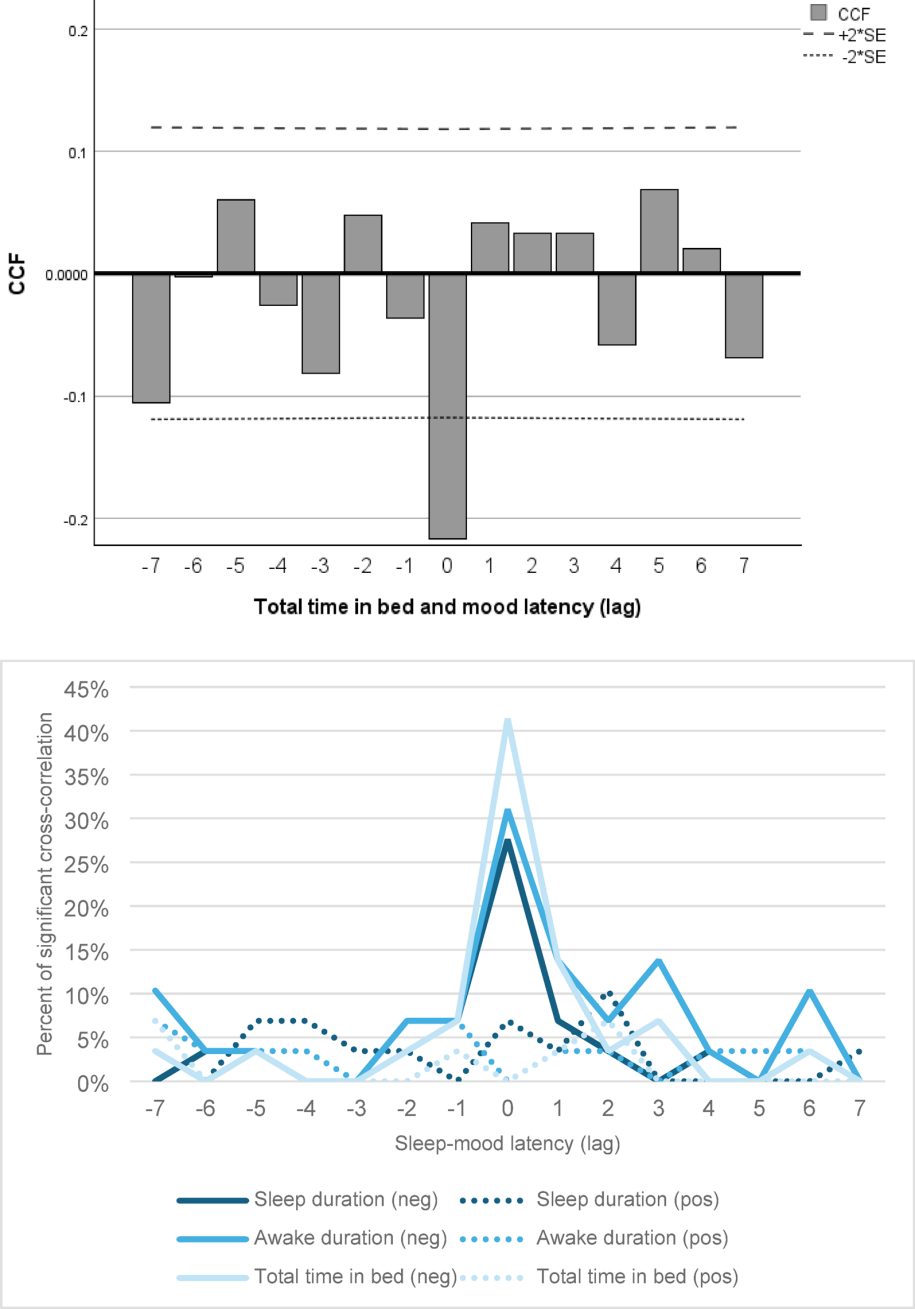
(**A-B**): Significant cross-correlation between sleep and mood change. **A**: Example of one CCF analysis for 1 participant for total time in bed, showing a pattern of spending more time in bed the night before a mood decrease, or spending less time in bed the night before a mood increase. **B**: Significant CCF of sleep duration, awake in bed duration and total time in bed and self-reported mood for all participants by time latency. For lag = 0, meaning that the sleep change occurred the night before the mood change, 28%, 31%, and 41% of all participants had a negative relationship between sleep duration, awake in bed duration, and total time in bed, respectively, and their mood. CCF=cross correlation function; SE = standard error; neg = negative CCF; pos = positive CCF. We used a line graph for illustrative purposes. Please keep in mind that the lag values on the x-axis are specific values and do not present a continuous process

The CCF analyses of sleep duration, awake in bed and total time in bed are presented in Figs. 1A-B. Figure 1A shows an example of the cross-correlations of total time in bed and mood, from one patient, as well as the boundaries of ± 2*SE. In this example, we see a negative correlation between total time in bed and mood change the following day. When this patient spent more time in bed, their mood became more depressed the following day, and when they spent less time in bed, their mood became more manic. Figure 1B presents an overall summary of the percentage of participants who exhibited either a negative or positive correlation between their sleep and mood, with a correlation coefficient greater than ± 2*SE.

The CCF analyses over the one-year period most frequently showed a negative relationship between unexpected changes in sleep duration, changes in time spent awake in bed, and changes in total time in bed the night before a mood change (lag = 0) (Fig. 1B). Specifically, 28% (*N* = 8) of participants showed a negative correlation between sleep duration and mood changes, 31% (*N* = 9) between time spent awake in bed and mood changes, and 41% (*N* = 12) between total time in bed and mood changes. This indicates that 28–41% of participants experienced an increase in sleep variables on the night before their mood scores indicated a shift towards depressive mood, or a decrease in sleep variables on nights before their mood scores shifted towards manic mood. Around two-thirds of the participants did not display a significant CCF between sleep changes and mood the following day (59%-72%).

To facilitate comparison with the findings reported by Bauer et al. (2006), we aggregated data from the two nights preceding a mood change (lags − 1 and 0). We found that 28% (*N* = 8), 34% (*N* = 10), and 41% (*N* = 12) of all participants had a negative CCF for either day between changes in sleep duration, awake in bed duration or total time in bed, respectively, and mood change. Participants were counted only once if they had significant correlations on both days.

Positive relationships, where changes in sleep duration, awake in bed duration or total time in bed were associated with a shift in mood scores in the same direction, were not common and found in no more than three participants on any given day (± 7 days of the mood change) (see Fig. 1B). In other words, there was no relation between increasing sleep indices and a shift towards manic mood (or vice versa for depression).

#### Fluctuations of sleep over 7 days and mood changes

**Fig. 2:**
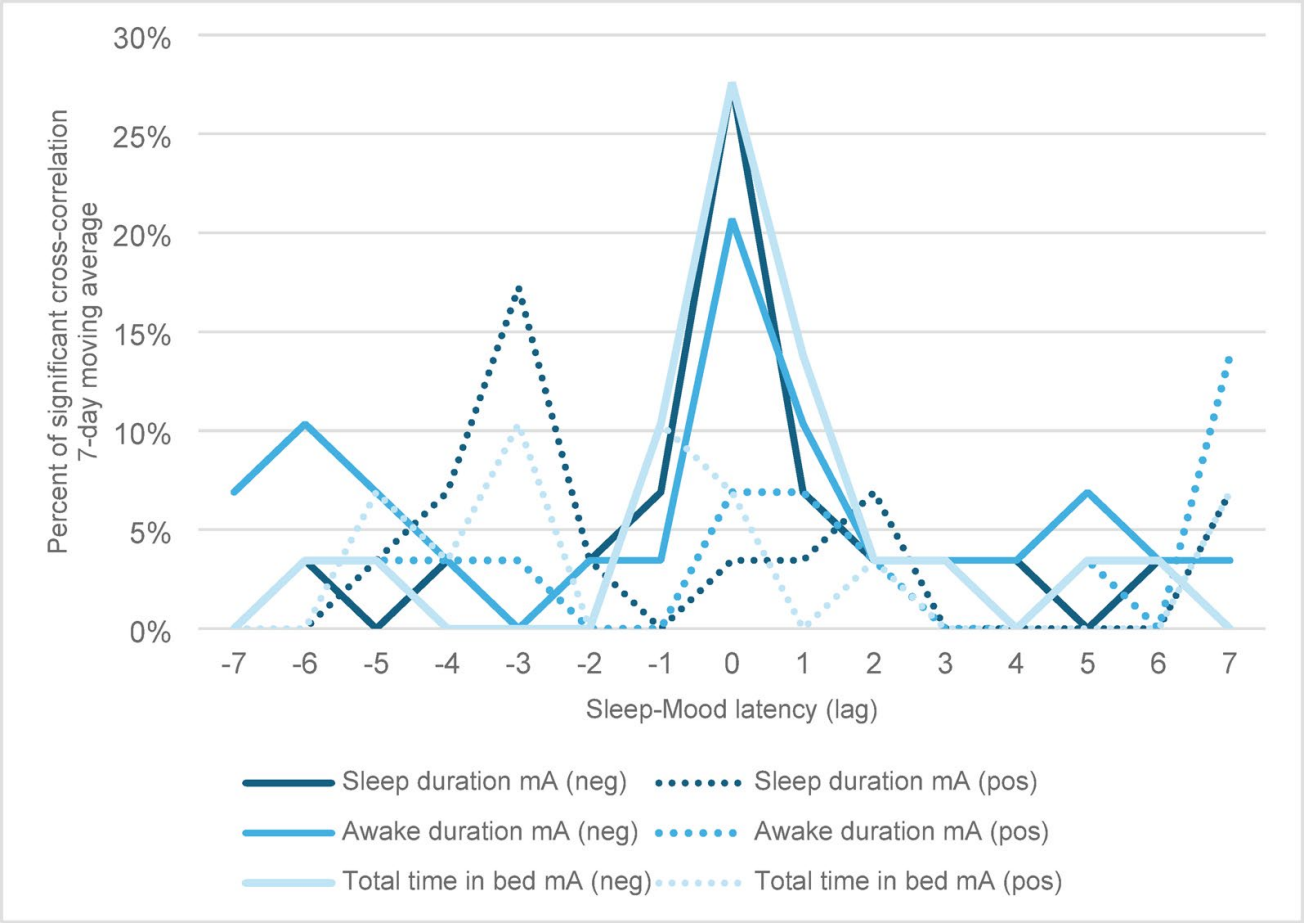
Significant cross-correlation function between 7-day moving average (mA) of sleep duration, awake in bed duration, and total time in bed, and mood for all participants by time latency. For lag = 0, meaning the average sleep change occurred over the 7 days preceding the mood change, 28%, 21%, and 28% of all participants had a negative relationship between sleep duration, awake duration, and total time in bed, respectively, and their mood. CCF = cross-correlation function; mA = moving average; neg = negative CCF; pos = positive CCF. Please keep in mind that the lag values on the x-axis are specific values and do not present a continuous process

The CCF analyses of the 7-day moving average *(mA)* sleep duration, awake-in-bed duration, and total time in bed are presented in Fig. 2. The Figure shows the percentage of participants who had a significant correlation between their 7-day average sleep and mood fluctuations.

The CCF analyses most frequently showed a negative relationship between the 7-day moving average of sleep and mood, with the most common correlation occurring within the seven nights preceding mood changes (lag = 0). Specifically, 28% (*N* = 8) of all participants experienced a negative relationship between the 7-day moving average (*mA*) of sleep duration and mood changes or of total time in bed and mood changes. Additionally, 21% (*N* = 6) of all participants had a negative relationship between *mA* awake in bed and mood changes for lag = 0. In other words, 21–28% of all participants experienced a decrease (or increase) in mood scores after experiencing an increase (or decrease) in their average sleep variables over the previous week.

#### Fluctuations of sleep variability over 7 days and mood changes

**Fig. 3:**
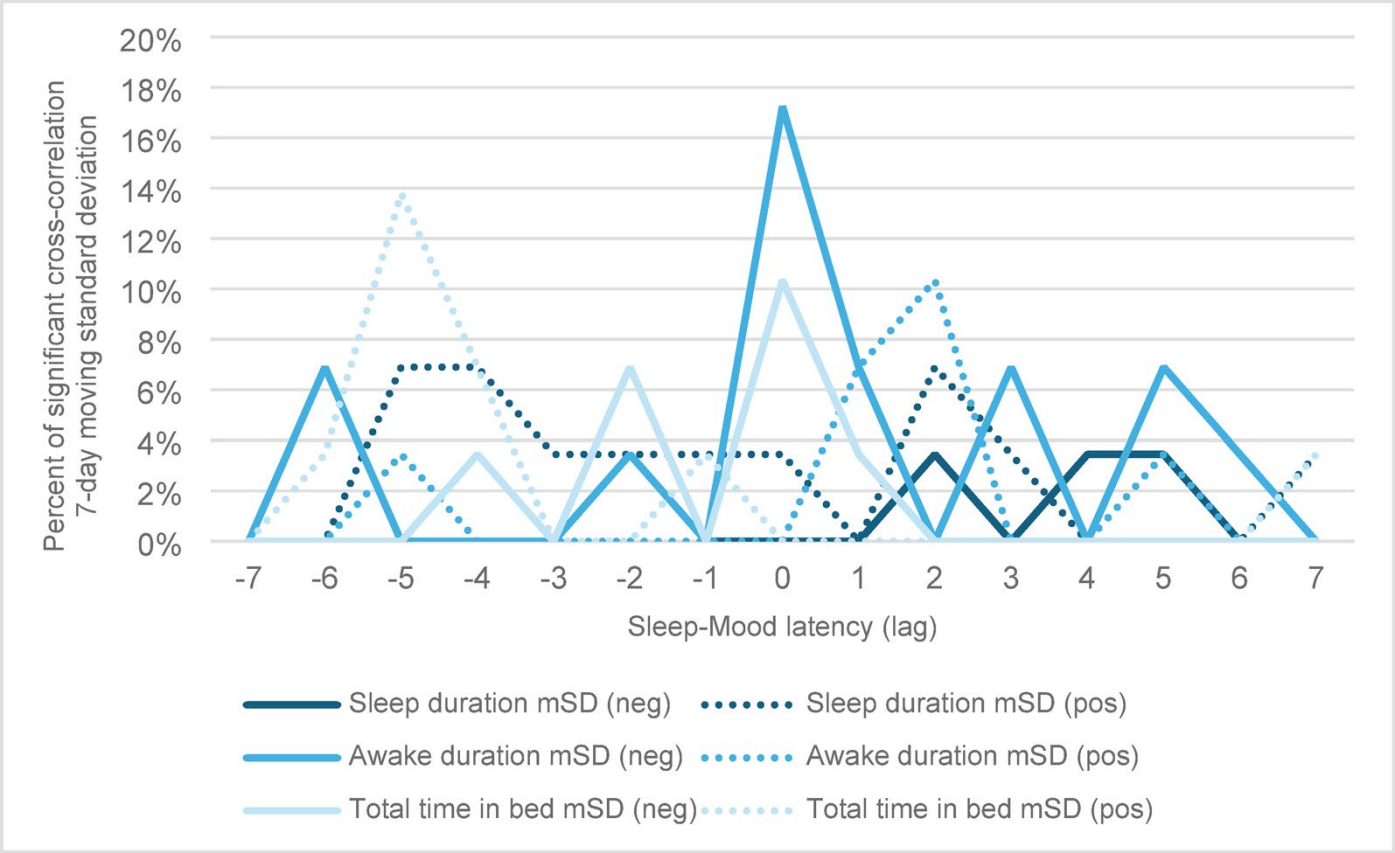
Significant cross correlation between 7-day variation (SD) of sleep duration, awake in bed duration and total time in bed, and mood for all participants by time latency. Few participants showed a linear relationship between sleep duration variability and mood, or total time in bed variability and mood. For lag = 0, meaning the change in sleep variability occurred over the 7 days before mood change, 17% had a negative CCF for variability of awake in bed duration, and 11% had a negative CCF for variability of total time in bed and mood. CCF = cross correlation function; mSD = moving Standard Deviation (of the previous 7 Days); neg = negative CCF; pos = positive CCF. Please keep in mind that the lag values on the x-axis are specific values and do not present a continuous process

The CCF analyses of the 7-day moving standard deviation *(mSD)* sleep duration, awake in bed duration and total time in bed are presented in Fig. 3. The Figure shows an overall summary of all participants and the percentage of participants who had a significant CCF for any given latency.

The patterns across these sleep variabilities are less pronounced, with fewer participants experiencing statistically significant correlations of changes in sleep variability over 7 days around their mood changes. Specifically, 17% (*N* = 5) and 10% (*N* = 3) had a negative relationship between *mSD* awake in bed and mood and between *mSD* total time in bed and mood, respectively, for lag = 0. In other words, these participants experienced an increase (or decrease) in the variability of their sleep variables (i.e. more fluctuations) during the 7 nights before their mood scores decreased (or increased).

## Discussion

This study validated and expanded on key findings from nearly 20 years ago Bauer et al. 2006), namely that there appears to be an inverse relationship between sleep changes and mood changes the following day in about one-third (28–41%) of BD patients. Although the two cohorts differ in sample size and study duration (the original study involved 59 participants with an average of 169 study days, compared to the present study with 29 participants and 364 study days), the study design, participant inclusion criteria, and data collection methods were almost identical.

### Relationship between fluctuations of sleep and mood

The most prevalent pattern observed for a single night was a negative relationship between changes in sleep the night before and changes in mood the following day (lag = 0), which was observed in approximately one-third of participants (28–41%). These participants experienced a change towards sleeping longer, spending more time in bed or lying in bed awake on nights preceding a shift toward depressed mood, or conversely, exhibited reduced sleep and time in bed before a shift toward manic mood. In contrast, this was much less common (7% for either sleep variable) for sleep changes two nights before a mood change (lag = -1). Overall, these findings suggest that some sleep patterns may serve as a prodromal indicator of mood changes in a substantial minority of individuals with bipolar disorder.

Some conclusions from other studies suggest that for BD, sleeping either for a short duration (less than 5 h) or a long duration (more than 10 h) may both be associated with depressive symptoms, compared to sleeping 7–8 h (Kaufmann et al. 2016). Furthermore, patients generally do not report changes in sleep duration before depressive mood (median 24% of patients report sleep changes preceding depression) (Jackson et al. 2003), and not all other studies find a significant relationship between sleeping more and experiencing more depressive symptoms the next day (Dominiak et al. 2022). However, some studies have found that self-reported sleep and mood, when measured on a scale from depression to mania with neutral in the middle, are negatively associated (Melbye et al. 2021)—similar to our findings—and that longer sleep reduces the likelihood of manic symptoms the following day (Leibenluft et al. 1996). Taken together, these findings may suggest some patient-specific differences in how sleep relates to particularly depressed mood, and the absence of a clearer trend than what was observed in our data might be due to the ambiguous correlation between sleep and depression. Interestingly, a large study of healthy adults (*N* = 2115) found that reduced total sleep duration was associated with more depressive symptoms (*p* < 0.001), on a day-to-day basis, and they reported that those who had more consistent sleep durations had improved mood (Fang et al. 2021). It would thus appear that those in our study who report a negative correlation between sleep and mood changes have the opposite correlation pattern as healthy adults. It may be that the negative correlations are more prominent when patients are experiencing more severe symptoms of bipolar disorder, or that those 1/3 of the patients with a negative correlation between sleep and mood change have other characteristics in common to indicate a more severe subtype of bipolar disorder. We did not further investigate this for this publication.

### Comparison to the original analyses

Key findings from the original analyses indicated that, for sleep the night before a mood change (lag = 0), the most common correlation was between total time in bed and mood change. Specifically, 34% (*N* = 20) showed a negative correlation between total time in bed and mood the following day, meaning they spent more time in bed before experiencing depressive symptoms and less time before manic symptoms (Bauer et al. 2006). When combining results for the two nights prior to a mood change, this proportion increased to 41% (*N* = 24). Our results similarly showed that the cross-correlation function (CCF) of total time in bed and mood was the most common correlation (41%), and it was also most pronounced on the night before the mood change (lag = 0). Combining lag = -1 and lag = 0 in our sample, the combined number of participants with a significant negative relationship on either or both days remained at 41%, as no participant demonstrated a correlation for lag = -1 but not for lag = 0. For the other sleep variables, when combining lag = -1 and lag = 0, the original analyses identified a negative correlation in 34% for sleep duration and in 22% for awake in bed duration. Similarly, our findings were 28% (sleep duration) and 34% (awake in bed duration). Overall, our findings support and confirm the results of the previous study, namely that changes in sleep variables - especially total time in bed - tend to precede and inversely correlate with mood changes in up to two-fifths of patients.

### 7-day average and variability in sleep changes and mood

To expand and identify potential new nuances in sleep patterns and mood correlations, we calculated a moving average (*mA*) and a moving standard deviation (*mSD*). These combined the values for the past 7 days for each day, by calculating the mean value of, for example, sleep duration over the last 7 nights (*mA*) and then the SD of that (*mSD*), in a continuous manner.

Approximately a quarter of patients (21–28%) exhibited a negative relationship between the 7-day average of the three sleep variables the week preceding the mood change (lag = 0). For *mSD*, aside from a negative relationship between *mSD* awake in bed and mood (17%, lag = 0) and a positive relationship between *mSD* total time in bed and mood (14%, lag = -5), very few participants demonstrated a significant relationship between sleep variability and mood change. Examining the standard deviation of sleep variables over 7 days does not seem to be an effective method for predicting mood shifts. However, the few results may also reflect the small number of participants, and a larger study may find that this pattern is valuable for some. Interestingly, when examining the relationship between the average sleep or sleep variability over the 7 days before a shift to a clinical mood episode from euthymia, it was sleep variability that proved to be the most reliable predictor, as reported in a previous publication of the same study population (Ulrichsen et al. 2025). One explanation may lie in the apparent fact that mood change does not necessarily coincide with the onset of a mood episode, and the link between sleep and either mood change or a mood episode might differ. Sub-clinical mood fluctuations could be more sensitive to immediate sleep changes, whereas fluctuations (variability) in sleep over multiple days may serve as an indicator or even a precursor of a change in BD episode or the onset of a BD episode.

This study examined changes in sleep variables and their potential correlation with changes in mood scores. It is important to distinguish between low or high mood scores and depressive or manic episodes. Patients may experience mood shifts without these changes reaching clinical significance. However, mood fluctuations could indicate a possible onset of a new clinical mood episode. A previous publication from the same cohort of the BipoSense study found that self-reported mood scores were significantly decreased two weeks before the onset of a depressive episode, as diagnosed by a clinical psychologist (Ludwig et al. 2024). A systematic review that investigated patients’ own identified prodromes to mood episodes found that mood change served as a prodrome for both manic and depressive episodes in nearly half of all patients (manic episode: median = 48%; depression episode: median = 43% )(Jackson et al. 2003). All our analyses are exploratory, and we cannot comment on causation. However, looking at the various sleep patterns and their relationship to both mood changes and episode onsets could potentially enhance our understanding of how sleep disturbances impact mood in bipolar disorder.

### Limitations

One of the main limitations of this study is the size of the cohort. The smaller sample size is somewhat countered by using daily data for one year, totalling 9,433 completed e-diaries. Given that we found similar results to those in larger cohorts, such as the ones in the original study(Bauer et al. 2006), the size of the cohort in this study may not have had a significant impact on the overall findings.

As we did not have diagnostic classification criteria varying daily, i.e. daily diagnostic interviews, we did not examine whether mood scores reached specific levels of mania or depression. We have also not made any classifications on severity of mood disturbances based on the self-reported scores, e.g. severe depression if scores where less than 20. The data shows trends towards the direction of change to either mood pole, but if the patient already had very high scores of either depression or mania, a shift towards the opposite pole would not necessarily involve displaying most of the symptoms associated with that pole (i.e., moving past the neutral score of 50). Furthermore, our results demonstrate the correlation between daily fluctuations in sleep and mood, but not whether this correlation was more common before the onset of manic or depressive symptoms. However, to achieve statistical significance over a year, after removing trends and patterns from the data, the correlations between sleep and mood would have had to be both high and consistent in both directions. Focusing on changes in mood direction, rather than specific mood scores, allows the findings to be applied more broadly and across everyday life, regardless of whether a patient is euthymic or experiencing a mood episode, as patients are presumably more likely to exhibit shifts in mood rather than switch entirely between episodes.

Using self-reports instead of objective sleep measures may introduce bias in reporting. People with BD seem to underestimate their sleep duration by nearly an hour, as reported in a study of 21 individuals with BD and 28 healthy controls (Ritter et al. 2016). This was significantly less common in healthy controls (*p* = 0.02). The study did not, however, find that patients with BD would incorrectly estimate their sleep latency, i.e., how long they lie awake before falling asleep. The authors acknowledge that their objective sleep measure, actimetry, may also underestimate actual sleep duration, as it is highly sensitive to movement detection. However, given the significant difference in estimated sleep duration between BD and healthy controls (healthy controls underestimated their sleep by less than 5 min), there may be a true tendency to underestimate sleep in BD. This is relevant when interpreting our results. Since all participants had BD, the estimated sleep duration might be underestimated; however, the intra-individual changes in sleep duration should remain consistent, thus not affecting the overall outcome. Furthermore, the ChronoRecord software is a well-validated tool for tracking and reporting symptoms in BD (Bauer et al. 2008, Bauer et al. 2004). Using a simple at-home method, which can be as basic as a pen and paper, enables these techniques to be scaled up in clinical practice, rather than requiring patients to use actigraphy devices or polysomnography for everyday sleep monitoring. Self–report is easily accessible, straightforward to use and understand, and cost-effective. Our key findings relate to changes in total time in bed, which may also be a more dependable measure, as it is generally easier to estimate the time getting into and out of bed than to pinpoint the exact moment sleep begins (Bauer et al. 2006).

There was an excellent compliance rate (89%) in filling out the daily e-diaries. However, patients were not able to fill out the data later if they had forgotten a day. The strict cut-off of 23:59 for reporting on the past day’s sleep and mood was implemented to avoid retrospective bias and to encourage reporting their data when it was most clear in their minds. However, there may be some missed days due to worsening of bipolar disorder, where patients may not have the energy or desire to comply with the study, or forget to fill out their e-diaries. Thus, in the missing 11% of the e-diaries, may lie valuable information regarding changes in mood and sleep correlations.

Finally, we have not included information regarding the medications the participants have taken throughout the year in the study. This data was self-reported and difficult to verify retrospectively and we have therefore not included it. Patients may differentiate based on the type and dose of medications they receive; however, it is beyond the scope of this paper to calculate such differences, and furthermore, we believe there is value in looking at the general patterns observed in people with bipolar disorder, regardless of the medication they may receive.

### Implications and future directions

Based on our findings and in comparison to previous results, at-home monitoring of changes in total time in bed may be beneficial for patients, alongside tracking of mood changes, to increase awareness of potential clinical changes and encourage help-seeking. The high compliance rate (89%) would indicate that patients find the reporting of sleep and mood data manageable and easy to use by the end of nearly every day.

This study reports on a small sample of BD outpatients. It would be interesting to test the findings with a larger sample size, potentially incorporating a feedback loop to see if the observed sleep changes can be stabilised or improved, and whether that affects mood outcomes. Promising studies are underway, such as the SBAA-BD study (Mühlbauer et al. 2018), which examine multiple potential early warning signs in BD and employ a smartphone system to detect behavioural changes and provide feedback to clinicians; however, they do not yet include sleep data in their protocol.

Finally, since our investigation focused solely on consistent changes in sleep and mood over a year, it would be valuable to conduct further analysis of the data using minimum thresholds for changes in both sleep and mood, as well as focus on the individual mood polarity. This could help identify the most common sleep-related precursors to mood fluctuations when mood changes become more severe or prominent, and determine which patients might benefit most from targeted monitoring and intervention.

## Conclusion

This study aimed to explore the relationship between sleep fluctuations and mood changes in BD, and to determine if findings from a similar study conducted nearly twenty years ago could be replicated. We observed a very similar pattern: 41% of participants exhibited a negative relationship between time spent in bed and mood the following day, meaning around two-fifths of patients spent more time in bed the night before their mood changes towards depression or less time in bed before a change towards mania. Building on the original research, we further analysed moving averages and variability of sleep over 7-day periods to expand upon the analyses presented previously. We found that while a few participants showed consistent changes in sleep variability around mood shifts, about one-quarter exhibited a negative relationship between their 7-day sleep average and mood prior to a mood change.

These results suggest that clear, predictable relationships between sleep patterns and mood fluctuations are not universal in BD, but prevalent in a large minority of patients. For them, specific changes in sleep variables, such as time spent in bed, may reliably precede mood fluctuations, particularly the night before. Nonetheless, most do not display consistent statistically significant patterns, highlighting the importance of personalised tracking and considering other potential markers.

## Data Availability

The datasets used and/or analysed during the current study are available from the corresponding author on reasonable request.

## Abbreviations

BD: Bipolar disorder
YMRS: Young mania rating scale
MADRS: Montgomery and Asberg Depression Rating Scale
ARIMA: Auto-Regressive Integrated Moving Average
CCF: Cross correlation functioning
SE: Standard error
SD: Standard deviation
mA: Moving average
mSD: Moving standard deviation
